# Supplementary material for: Content-rich biological network constructed by mining PubMed abstracts
Source: BMC Bioinformatics. 2004 Oct 8;5:147. doi: 10.1186/1471-2105-5-147 (PMC528731; doi:10.1186/1471-2105-5-147)
Supplement: Additional File 2 — The original results of the above study (non-essential files are deleted to keep the file size under the limit set by BMC bioinformatics). [file 1471-2105-5-147-S2.bz2 › chilibotAdditionalFile2/dip10/125ID9261098E469/html/YWHAZ_RAF1.html]

 


 **YWHAZ** and **RAF1** 
  
Found 34 abstracts in PubMed, retrieved 10.  
 

 What does Google say? 
 PDF only 
| .edu only 

---

**Interactive relationship** (e.g. stimulation, inhibition, etc)

**Neutral relationship**- Here we report that thymic lymphomas overexpressing Ras and Raf 1  [ **RAF1** ]  exhibit low expression of Fas  [ **YWHAZ** ] .  Ref: 12389631 Leuk Lymphoma, 2002
- Changes at genomic, transcriptional and post translational levels of G proteins and protein kinases Ras, B Raf  [ **RAF1** ]  and their transcription factor effectors c Jun, ATF2, Stat3 and NF kappaB affects TNF, Fas  [ **YWHAZ** ]  and TRAIL receptors, which play important roles in acquiring melanoma s resistance to apoptosis.  Ref: 12789291 Oncogene, 2003
- Furthermore, Raf 1  [ **RAF1** ]  deficient fibroblasts are more sensitive than wild type cells to specific apoptotic stimuli, such as actinomycin D or Fas  [ **YWHAZ** ]  activation, but not to tumor necrosis factor alpha.  Ref: 11296228 EMBO J, 2001
- Fas  [ **YWHAZ** ]  recruited FLIP interacts with TNF receptor associated factors 1 and 2, as well as with the kinases RIP and Raf 1  [ **RAF1** ] , resulting in the activation of the NF kappaB and extracellular signal regulated kinase Erk signaling pathways.  Ref: 10837247 Curr Biol, 2000
- 4 The up regulation of Fas  [ **YWHAZ** ]  and down regulation of Bcl 2 were observed in osteoblasts expressing H Ras V12, H Ras V12 T35S, or Raf 1  [ **RAF1** ] .  Ref: 11934900 J Biol Chem, 2002
- Whereas the deoxycholate induced increase in Mcl 1 reduced Fas  [ **YWHAZ** ]  mediated apoptosis, the Raf 1  [ **RAF1** ]  inhibitor potentiated Fas  [ **YWHAZ** ]  apoptosis.  Ref: 12438243 Cancer Res, 2002

**Non-interactive relationship** (e.g. studied together, co-existance, homology, etc.)

- Thus, we propose that H Ras signals followed by Raf 1  [ **RAF1** ]  MAPK pathway but not PI3K not only reduces beta 1 mediated adhesion of osteoblasts to matrix proteins but induces apoptosis presumably via the Fas  [ **YWHAZ** ]  up regulation and Bcl 2 down regulation.  Ref: 11934900 J Biol Chem, 2002
- Published results implicate PI3 kinase as a target of oncogenic Ras activity leading to the suppression of Fas  [ **YWHAZ** ]  but whether other Ras targets e.g. Raf 1  [ **RAF1** ]  are also involved is unclear.  Ref: 12389631 Leuk Lymphoma, 2002
- These observations provide evidence for involvement of Raf 1  [ **RAF1** ]  MEK ERK pathway in Ras mediated inhibition of Fas  [ **YWHAZ** ]  expression and in selective promotion of survival of lymphoma cells.  Ref: 12389631 Leuk Lymphoma, 2002
- We show that expression of Fas  [ **YWHAZ** ]  in these lymphomas can be increased not only in the presence of a specific inhibitor LY294002 of P13 kinase, but also in the presence of specific inhibitor PD98059 of MEK, downstream target of Raf 1  [ **RAF1** ] .  Ref: 12389631 Leuk Lymphoma, 2002
- Although normal spreading onto fibronectin was restored by the beta 1 integrin affinity activating antibody TS2 16 in cells expressing FA Csk or Raf  [ **RAF1** ]  BXB, FAs  [ **YWHAZ** ]  were lost in these cells.  Ref: 11809811 Mol Cell Biol, 2002
